# Work, Self, and Society: A Socio-historical Study of Morita Therapy

**DOI:** 10.1007/s11013-024-09845-9

**Published:** 2024-02-19

**Authors:** Yu-Chuan Wu

**Affiliations:** Institute of History and Philology, Academia Sinica, Nangang 115, Taipei, Taiwan

**Keywords:** Morita therapy, *Shinkeishitsu*, Neurasthenia, Work therapy, Work efficiency

## Abstract

Morita therapy is known as a psychotherapy grounded in the culture of Japan, particularly its Buddhist culture. Its popularity in Japan and other East Asian countries is cited as an example of the relevance and importance of culture and religion in psychotherapy. To complement such interpretations, this study adopts a socio-historical approach to examine the role and significance of work in Morita’s theory and practice within the broader work environment and culture of the 1920s and 1930s in Japan. Morita conceptualized *shinkeishitsu* as a personality disease and a social illness caused by an alienating work environment. He proposed a remedy that emphasized the subjective emotional experience of work. To his primarily middle-class clients and readers, Morita’s reconciliation between the self and society and that between autonomy and compliance was persuasive and useful, providing a philosophy whereby they could integrate into the work environment without loss of self-worth. The socio-historical character of Morita therapy is vital to understanding its power and appeal during Morita’s time. Moreover, it sheds light on the complex interrelationships between work, mental health, and society.

## Introduction

Morita therapy, an inpatient psychotherapeutic program developed by the Japanese psychiatrist Morita Masatake (1874–1938), has been identified as a form of psychotherapy rooted in the culture of Japan, particularly its Buddhist culture (Chervenkova, 2017; Fujita, 1986, pp. 41–53). Beginning with Morita, who often employed Buddhist terminologies to explain his ideas, generations of Morita therapists have resorted to Buddhist teaching on ego fixation and ego renunciation to elucidate and expand on Morita’s work, and compared the transformative insights obtained through Morita therapy to the spiritual enlightenment pursued by Buddhism, particularly Zen cultivation (Kitanishi, 2001; Levine, 2018; Okamoto, 2015; Reynolds, 1989; Usa, 1987). The cultural and spiritual features of Morita therapy are considered key to its success in Japan and other East Asian countries (Cui, 2004; Miao, 1993; Min, 2003), where, religious scholar Shimazono Susumu argues, Morita therapy has been embraced as a remedy for the spiritual crisis engendered by the antagonism between science and religion (Susumu, 2003).

To complement such interpretations, this paper attempts a socio-historical analysis of Morita’s original therapy by focusing on the roles and significance of work. Work, as we shall see in this paper, was not only the main therapeutic tool in Morita’s program, but also a major discussion topic between Morita and his clients. Many of Morita’s clients’ interests, concerns, and obsessions centered around work; Morita, in response, had shared his thoughts on work from a wide range of perspectives, ranging from physiological and psychological to familial, social, and political. This reflected the significance of work in the contemporary Japanese society, as work, then, was a core political, economic, social, and cultural issue and constituted a vital part of a person’s self-identity and self-esteem as well as social, familial, and interpersonal relationships. Hence, work provides a window for historically understanding Morita therapy and its power and appeal in Morita’s time. Moreover, Morita’s use of work in psychotherapy, which aims to restore clients’ agency in working and everyday life and help them embrace the real working environments, may shed some light on the complex interrelationships between work, mental health, and society.

From moral treatment to occupational therapy, work has been a mainstay treatment in modern psychiatry, with the ability to work and be a productive member of society regarded as a key indicator of mental health and a central goal of psychiatric treatment. Numerous treatment programs and rehabilitation and recovery schemes have been developed to train mentally ill persons’ work ability, improve their social skills in the workplace, help them cultivate the attributes deemed necessary for a successful working life, and, ultimately, reintegrate them into society. However, work therapy programs have often been based on the dominant assumptions about work, as well as personhood and citizenship, of their times, which, while restricting patients’ experience with work within therapy, have often contributed to their illness in the first place. Being a pioneer in work therapy in Japan, Morita has come to realize the problems with the conventional work therapy and developed a therapeutic model that focuses on the subjective emotional experience of work. As we can learn from the extensive exchanges between Morita and his clients, much of their suffering originated from their obsession with the contemporary ideologies of self-made success and associated work morals and ethics, which had become increasingly impractical in a rapidly industrialized and bureaucratized working environment. The role of work in their mental illness was reflected in the fact that, before seeing Morita, most of them were diagnosed or self-diagnosed as neurasthenia, a quintessential disease of overwork. To help them transcend those intellectualized and socially derived obsessions, Morita devised his work therapy based on a logic of emotion, through which clients could come to experience work in a refreshingly immediate and intuitive way. By immersing themselves in the present situations, they would also learn from experience about the interpersonally, socially, and environmentally situated nature of work. This helped them adapt to and embrace the work environment without loss of self-initiative and self-worth. Therefore, reconsidering Morita therapy from the perspective of work therapy can enrich our understanding of the role of work in mental treatment and contribute to the critical study of contemporary occupational therapy and rehabilitation. It also sheds light on the social and historical features of Morita therapy, which may provide clues for the continuous development and relevance of Morita therapy in an era in which work has lost much of its cultural and moral significance.

## Work and Morita Therapy

Morita’s residential treatment program comprised four stages: (1) isolation–rest therapy; (2) light work therapy; (3) heavy work therapy; and (4) complicated activity therapy, in preparation for real life. Counting reading as work, the last three stages represented effective work therapy, in which the clients were asked to constantly engage in work. Work was also an implicit and important theme in the first stage, with its completion contingent on the increase in clients’ desire for work. Using his residence for inpatient treatment, Morita had his patients perform daily household chores. He neither prepared or designed tasks nor established a strict schedule. Instead, he asked the clients to pay attention to their surroundings to find tasks that needed to be performed in a household. This distinguished his regime from other contemporary work therapies, which Morita criticized as too mechanical and artificial and reducing work to a formality (Morita, 1974b, pp. 348–361).

Work featured prominently in Morita’s clients’ concerns and suffering. Nearly all his inpatients were diagnosed with *shinkeishitsu*.^1^ Most of them belonged to affluent families who could afford a month-long stay. Among them were university or prospective university students, rich farmers, military and government officials, teachers, businesspeople, salaried individuals, small shop owners, and educated housewives (Morita, 1974b, pp. 266–273). While they apparently experienced the symptoms of *shinkeishitsu*,^2^ many harbored work-related anxieties. Students were anxious about high school or university entrance examinations and vocational choices. Employees were concerned about work performance, workplace relationships, promotion prospects, and job security. Even housewives cared about their household roles and responsibilities. These concerns were inseparable from their experiences with *shinkeishitsu*, to which their failure was typically attributed. Furthermore, some *shinkeishitsu* symptoms were explicitly and directly related to work. They included general complaints, such as attention and memory difficulties that affected study and work performance, and a few specific syndromes, such as erythrophobia (*sekimen kyōfushishō*) and scopophobia (*shisen kyōfushishō*), which arose out of concerns about interpersonal relationships at school and the workplace (Morita, 1974b, pp. 186–264), and writer’s cramp (*shokei*), which was related to the increased load of office paperwork (Morita, 1974c, pp. 137–157).

Work was a major theme in *Keigai Kai*,^3^ a monthly gathering of former and current inpatients who shared their experiences and received Morita’s instructions. Work and study efficiency were among the most common discussion themes (Morita, 1974e, p. 757). Other recurring work-related themes included the regime of work and rest, career choices, workplace relationships, labor disputes, and Marxism and Communism. Many clients shared that Morita therapy improved their work performance and workplace relationships, which motivated them to continuously participate in *Keigai Kai* even after discharge.

Notably, Morita’s use of work in psychotherapy had evolved over time. Before inventing new roles and functions of work in psychotherapy, he had adopted conventional models of work therapy in contemporary psychiatry. This evolving process provides important clues for understanding Morita’s theory and therapy of *shinkeishitsu*.

## Work and Contemporary Japanese Psychiatry

Morita’s experience with work therapy can be traced back to the early twentieth century when he was training to become a psychiatrist at Sugamo Hospital, the mental hospital of Tokyo Imperial University. There, he was asked by his mentor, Kure Shūzo, to organize and take charge of work therapy (*sagyō ryōhō*) for hospitalized patients (Morita, 1974b, pp. 407–408). Kure studied under Richard von Krafft-Ebing and Emil Kraepelin in Germany before serving as a long-term Chair of Psychiatry at Tokyo Imperial University. He is known as the “founder of Japanese psychiatry” for his contribution to the introduction and implementation of Western, particularly German, psychiatry and the modernization of the custodial care of mental patients in Japan. As the superintendent of Sugamo Hospital, Kure famously abolished the use of chain and shackle and replaced them with more humane treatment measures in line with Western mental hospital standards, including work therapy (Okada, 1982, 2015, pp. 163–176).

Work therapy developed in Western Europe and North America during the nineteenth century and constituted the cornerstone of moral treatment, which sought to transform the mental hospital into a psychotherapeutic tool (Ernst, ed., 2016; Mohri, 2011). In the early stages, Kure and Morita largely followed the Western models of work therapy. Theoretically, they primarily regarded work therapy as a diversion therapy aimed at drawing patients’ attention away from pathological thoughts and emotions by engaging them in work. Moreover, it was used to rebuild patients’ character by enhancing self-confidence, strengthening their will, and fostering positive feelings, while training and rehabilitating their physical, mental, and social capacities. Practically, they stressed that doctors must carefully determine the types and amounts of work according to patients’ conditions, education, and former jobs and closely monitor patients’ work (Kure, 1916, pp. 368–385; Mohri, 2011). The occupations that Morita introduced at Sugamo Hospital included weaving, wallpapering, land cultivation, farming, and husbandry (Morita, 1974b, pp. 407–408).

After leaving Sugamo Hospital in 1906, Morita practiced at a private mental hospital (Negishi Hospital) while seeing outpatients at his home. During this period, the number of patients diagnosed or self-diagnosed with neuroses, primarily neurasthenia and hysteria, began to increase rapidly in Japan (Sato, 2013). Apart from inpatients with psychosis, Morita employed work therapy to treat neurotic patients. In particular, Morita was attracted to Otto Binswanger’s residential treatment regime for neurasthenic patients, which he called “the method of regulated living” (*seikatsu seiki hō*), and attempted its rigorous schedule of daily activities, of which work formed a substantial part, for the treatment of neuroses. To perform this treatment, he either admitted patients to Negishi Hospital or delivered such treatment during the day at his home, where he had the required equipment and material for certain occupations, such as carpentry (Morita, 1974a, pp. 576–579, 1974b, pp. 408–409).

However, when Morita finally established his “special treatment for *shinkeishitsu*” in the late 1910s (Morita, 1974a, p. 380), his work therapy was considerably different from that practiced at Sugamo Hospital and Binswanger’s “method of regulated living.” He deliberately replaced designed occupational activities with everyday household tasks and allowed his patients much more autonomy. Most importantly, Morita developed a new theory of work therapy that focused not on diversion, confidence-boosting, character-building, or skill training but on patients’ innate desire for and their immediate experience of work. This transformation was gradual and occurred through his encounters with a group of patients characterized by their intense concern for self-improvement and self-advancement, that is, the *shinkeishitsus*. Morita developed his work therapy to heal the *shinkeishitsus*’ anxieties, difficulties, and frustrations at work, which were driven by the contemporary work environment and culture as well as their personality traits.

## From *Risshin-Shussei* to a Bureaucratic Society

Morita’s clients lamented about their shattered hopes of *risshin-shussei*, regretted their weak character, and expressed fears of being left behind in the “struggle for survival.” These were important themes in the social and cultural history of Meiji Japan (1867–1912). A catchphrase in the Meiji era, *risshin-shussei* (establishing oneself and making progress in the world), the aspiration for self-advancement, inspired generations of Meiji Japanese to pursue personal success through assiduous study, political participation, and diligent work. Initially an ethos specific to youth from the families of former samurais and rich farmers and merchants, it spread to all levels of society and stirred people from lower social ranks and rural regions to strive for “success” (*seikō*) and climb up the social ladder (Kinmonth, 1981; Takeuchi, 1991, 1997; Wasaki, 2017). However, apart from the appeal of success, Meiji Japanese individuals’ efforts for personal advancement were also driven by the wretchedness of failure, as conceived by the principles of Social Darwinism that had become prominent in Japan since the mid-Meiji period. With social life envisioned as a struggle for survival, the threat of defeat and annihilation imposed a constant pressure on people to improve and seek educational and career advancement (Kinmonth, 1981). At the time, a person’s success or failure was believed to be contingent on their character. This belief was preached and spread by the best-selling book, *Saigoku Risshi Hen*, Nakamura Masanao’s translation of Samuel Smiles’ *Self Help*. The book greatly influenced Meiji youth, and its inspiring stories of self-made success instilled the belief that the virtues of industry, thrift, perseverance, and courage would lead them to realize their dreams; it implied that people only had their weak characters to blame for their failure (Kinmonth, 1981; Tajima, 2016; Tsutsui, 2009; Wasaki, 2017).

However, since the turn of the century, a growing number of people had begun questioning the merit and worth of their efforts and felt disillusioned and disconnected (Kinmonth, 1981; Wasaki, 2017; Watarai, 2003). The prospect of *risshin-shussei* and *seikō* became much less promising, if not discouraging and gloomy. It was increasingly difficult and competitive to advance in the educational system. With the establishment of a stable political and social order in the late nineteenth century, more people were committed to pursuing higher education for a better future. High school and university entrance examinations saw fierce competition. An identity and subculture characterized by a mixture of hope and despair, and optimism and skepticism, developed among the applicants (Takeishi, 2012; Takeuchi, 1991; Wasaki, 2017). Such students constituted a substantial share of Morita’s clientele and readership.

Meanwhile, the prospects for higher education graduates became more uncertain and gloomy. After the wars with China and Russia, industrialization, urbanization, and capitalism gathered pace in Japan. In particular, during and immediately after the First World War, Japan enjoyed a period of unprecedented economic prosperity. Economic growth was accompanied by corporation expansion, production mechanization, management bureaucratization, higher living costs, greater economic inequality, and the boom-and-bust cycle. Those who had pursued higher education with the *risshin-shussei* dream of achieving fame and wealth faced a highly competitive job market and were often resigned to mediocre and routine work, modest salaries, and no real responsibility. Job promotions were incremental and slow. Moreover, economic downturns threatened financial and job security (Kinmonth, 1981; Takeuchi, 1997).

Hence, following the turn of the century, the grand *risshin-shussei* dreams became increasingly impractical and unrealistic. When Morita’s clients expressed frustration at being unable to pursue their dreams because of illness, their aspirations included smaller successes rather than grand ambitions. For people, work had become a less potent source of meaning and identity, with growing feelings of alienation and insecurity among the working and middle classes. Amid declining social mobility and growing economic inequality, Marxism and Communism, as a body of thought as well as a social and political movement, began attracting greater interest and were viewed by the government as dangerous sources of social and political unrest. Meanwhile, more people turned their attention away from the external world and toward their inner selves. The resulting culture of self-cultivation saw the emergence of a host of physical and mental cultivation methods, some of which attracted a large following (Kitamura, 1998; Tajima, 2016; Takeuchi, 1997; Tsutsui, 2009; Wasaki, 2017). On the one hand, these self-cultivation practices, as a method of experiencing, exploring, and enriching one’s body and mind, afforded a sense of well-being and accomplishment and healed those who felt frustrated and dissatisfied with their life and career. On the other hand, those who sought advancement in the real world found such practices to be a means of self-enhancement that could improve their chances of success.

All the above themes relating to contemporary work culture feature prominently in the exchanges between Morita and his clients and in Morita’s writings. Morita’s clients felt the pressure of fierce social competition and expressed frustration at the bureaucratization, fragmentation, and trivialization of their work. Before receiving Morita’s therapy, many had tried a variety of self-cultivation methods. Some younger student clients, who were interested in literature and the arts, were asked by their parents to study more practical subjects and felt confused about their career choices. A few clients were attracted to Marxism and Communism, which became a recurring topic of discussion at Morita’s place (Morita, 1974e).

However, despite their disappointment, many of Morita’s clients were anxious to assimilate into the work environment. This was reflected in the frequency with which the topic of work efficiency (*shigoto nōritsu*) came up in their discussions (Morita, 1974e, p. 757). At the time, with industrial expansion, enhancing workers’ efficiency and productivity became an issue of great concern. With the advent of Frederick Winslow Taylor’s Scientific Management from the United States, employers, scholars, and government officials made collective efforts to implement its methods of analyzing, optimizing, and standardizing production processes and enhance public awareness about the importance of work efficiency (Hashimoto, 2001; Sasaki, 1998; Sasaki, et al., 1990). At the same time, a few scholars from the psychology and medicine disciplines were devoted to establishing a new field of study, namely, the Science of Labor, aspiring to provide a scientific and positivist approach to determining the most efficient form of production through laboratory studies of the psychology and physiology of work and fatigue (Furusawa, 1998; Mitsui, 1998; Sato, 2002, pp. 488–505).

Despite differences in methodology and perspectives on labor issues, both Scientific Management and the Science of Labor took an objective analytic approach toward work and fatigue and were aimed at their quantitative understanding and management. Such an approach and the resulting standardization, atomization, and depersonalization of the production process risked the further alienation of workers. Nonetheless, the concept of work efficiency were widely disseminated and associated with personal merit and competence. This is evident from Morita’s clients’ eagerness to improve their study and work efficiency, which also lay at the root of their disease. Concerning work, Morita’s clients were caught in the gap between social ideals and personal experiences and between social reality and personal aspirations. They strove to fulfill social ideals and advance themselves in society; however, they were inevitably dissatisfied owing to the unattainability of those ideals and aspirations. The gap between the ideal and reality, inherent in contemporary work culture, was central to Morita’s understanding of the pathology of *shinkeishitsu*.

## *Shinkeishitsu*: A Disease of Modern Civilization

As is widely acknowledged, Morita made an important contribution to the demise of the concept of neurasthenia as a neurological disorder in Japan by providing a psychological explanation of its symptoms and recasting it as *shinkeishitsu* (Katou, 2011; Junko, 2012, pp. 63–65, 2014, pp. 87–100). Morita refuted the then-popular notion that neurasthenia was a disease of nervous exhaustion attributable to the pressures of modern civilization. He argued that most of the so-called neurasthenic symptoms were subjective and caused by a psychological process called “psychological interaction” (*seishin kōgo sayō*), in which attention and sensation reinforced each other in a vicious circle to heighten sensitivity to certain “symptoms.” This psychological interaction, in turn, originated from an innate psychological disposition characterized by strong hypochondriacal tendencies, that is, *shinkeishitsu* (Morita, 1974b, pp. 284–298). Thus, Morita dismantled the idea of neurasthenia as a symbol of modernity and turned it from “a disease of overwork” to “a disease of personality” (Junko, 2012, pp. 63–65).

However, it should be noted that Morita did not conceptualize *shinkeishitsu* as a disease solely attributable to psychological disposition but emphasized its social origin. In a sense, Morita still regarded *shinkeishitsu* as a disease of modern civilization, resulting from the maladaptive reactions of a particular personality type to certain aspects of modernity. For example, he indicated that the advancement of modern medicine, combined with the rapid circulation of information and the market economy in modern society, was an important factor contributing to the increased prevalence of *shinkeishitsu*. According to him, modern medicine had amassed immense knowledge about varied diseases, which, through modern advertising, had been irresponsibly disseminated among the public by unscrupulous doctors and drug companies competing for patients/customers and a rapidly expanding profit-driven publishing industry. This unduly heightened people’s health concerns. Owing to their strong hypochondriacal tendencies, the *shinkeishitsus* were particularly susceptible to inaccurate and inappropriate health knowledge and were easily trapped into the vicious circle of “psychological interaction” of fear, hypervigilance, and hypersensitivity (Morita, 1974b, pp. 71–75).

Regarding the perils of modern life, Morita was most concerned about its adverse influence on how people lived their productive life and criticized the modernity of contemporary work culture, which he considered to be at the root of the *shinkeishitsus*’ misconceptions. Based on Morita’s writings and remarks, his comments can be summarized under the following four points: commoditization of work, homogenization of desire, objectification of work by the sciences of work and management, and contrived work morals promoted by the self-cultivation culture.

First, regarding the commoditization of work, Morita lamented that although equality and freedom had become watchwords in Japan after the abolition of the feudal system in 1868, people had, in effect, become more unequal and enjoyed less freedom. Purporting to put everyone on an equal footing, capitalism created far greater inequalities in wealth and power between the classes than the old feudal society. Moreover, despite the claim that individuals were free to choose their occupations and pursue personal advancement, capitalism molded everyone into the same shape by imposing a value system in which everything was appraised in monetary terms (Morita, 1974g, pp. 231–232). In contrast to the “age of rampant military power” of the feudal times, Morita called the capitalist period “an age of rampant monetary power,” in which work meant nothing more than a means of money-making (Morita, 1974g, pp. 406–421). He illustrated this attitude by referring to the new, imported term for office workers, *sararīman* (salaryman), contending that, in contrast to the traditional term *hōkōnin* or *hōshinin* (serviceman), which emphasized work or service, the new term implied a sole focus on wages (Morita, 1974g, pp. 536–537; 1974e, pp. 165, 520). Viewing work as a commodity, people, thus, detached themselves from work and were unable to experience it in personal and authentic ways.

Second, Morita argued that the rising consumerist culture had forced people’s wishes into a uniform mold and alienated them from genuine desires and needs. He was critical of the then-popular pursuit of “cultural life” (*bunka-seikatsu*), that is, a rational and scientific lifestyle based on the use of modern household appliances and items, which Morita considered symptomatic of people’s obsession or “intoxication” with material wishes. Morita blamed this obsession on a mixture of advanced advertising techniques, people’s vanity, and the ostentation of the capitalist class. In particular, he warned that the excess and extravagance of the newly rich (*narikin*) would have a strong suggestive effect and could become a major source of social discontent and resentment. Drawing on the byword of Marxism and Communism at the time, “dangerous thoughts” (*kikenshisō*), Morita described the ostentation of the rich as “dangerous acts” (*kikenkōi*), claiming that they were as harmful and hazardous as left-wing thought (Morita, 1974g, pp. 231–232, 265–267; 1974f, pp. 167–174).

Third, Morita blamed his clients’ obsession with such issues as work planning, schedule, and efficiency on the objectification of work in the industrial culture. He indicated that these issues were originally proposed concerning the management of industrial production and might have been helpful for productivity enhancement in large-scale factories, as the workers therein were subjected to mechanized and standardized production processes and deprived of autonomy and spontaneity. However, he was skeptical of the worth and effectiveness of these ideas beyond industrial production and expressed concerns about them being embraced by people across all occupations as guidance and work standards. In his view, the contemporary work sciences studying these issues considered work in mere objective terms and failed to sufficiently consider subjective perspectives and real work experiences. Owing to their conscientious and perfectionist tendencies, the *shinkeishitsus* were often keen to adopt these man-made, apparently rational, and objective ideals to guide and monitor their work. Consequently, they took a formalistic approach to work at the expense of spontaneity and creativity and were easily distressed by the inevitable gap between the ideal and reality (Morita, 1974b, p. 74; 1974g, pp. 386–397; 1974d, p. 581; 1974e, pp. 342, 626–633).

Finally, Morita condemned the contemporary culture of self-cultivation as another major source of the *shinkeishitsus*’ obsession. As stated above, self-cultivation, as a healing and self-improvement practice, attracted a great deal of interest. Before receiving Morita’s treatment, many of his clients had tried varied cultivation methods and were avid readers of popular cultivation books. In Morita’s view, most of the cultivation methods, including popular ones such as Okada Torajirō’s quiet-sitting, were ineffective because they only offered training in particular settings and were detached from real-life situations. He emphasized that the only effective way of self-cultivation was to train oneself by engaging in real work (*jijyō no tannen*). Moreover, he criticized the cultivation methods and books as harmful, as they taught morals and virtues in abstraction and promoted unrealistic expectations, irrespective of the nature and reality of the mind. Like scientific ideas about work, such moral ideals constituted a rigid framework, which shaped and restricted people’s work experiences and became a major source of anxiety, leading the *shinkeishitsus* into a vicious circle of introspection and self-criticism that reinforced and perpetuated their “symptoms” (Morita, 1974a, pp. 596–610; 1974e, pp. 266, 405–407, 438–440, 517–520, 560–561, 569–571, 645–654).

Furthermore, Morita was critical of both left- and right-wing thoughts and movements that were rising in Japan. In his view, Marxism and Communism were not really progressive; rather, like their right-wing counterparts, such as nationalism and fascism, they grew as a reaction against capitalism and capitalist culture. In fact, they shared the same ideologies as their supposed enemies and similarly fought for power and wealth by wielding weapons of thought (Morita, 1974g, pp. 268–273, 406–421, 495, 524–528; 1974e, pp. 144, 171–172). Owing to their tendency toward intellectualization, the *shinkeishitsus* was easily attracted to the contrived ideals of Marxism and Communism; however, they were different in nature from those reckless revolutionaries, who Morita believed were mostly hysterical or weak-willed degenerates motivated by fetishistic desires. The *shinkeishitsus* refrained from impudent and violent actions because of their scrupulous and conscientious natures and soon became disappointed and disillusioned (Morita, 1974d, pp. 95, 126, 184–185, 464–466; 1974e, pp. 144, 349–351).

Hence, while Morita conceived *shinkeishitsu* as an innate personality type, he highlighted the social origins of the *shinkeishitsu* disorder. The *shinkeishitsus*’ suffering originated in the contemporary ideologies about work and self as well as their psychological disposition. They were captured by those ideologies (*toraware*), which shaped and restricted their work experience, and suffered from the contradiction between ideas and reality (*shisō-no-mujun*). Conventional work therapy, which was based on idealized notions of what a good worker should be, was of little help and might even reinforce and perpetuate their obsessions. To free them from the intellectual traps, Morita emphasized the subjective and emotional aspects of work. He set up a systematic program to facilitate the clients to direct attention toward the immediate surroundings. Thereby, they could reconnect with their primal desire for work and learn to realize it in the real world by embracing the full physical, mental, and emotional reality of work.

## The Desire for Work

Morita regarded *shinkeishitsu* essentially as an anxiety disorder, manifested by the health anxiety of simple *shinkeishitsu*, fear of impending death of paroxysmal neurosis, and anxiety about specific repetitive intrusive thoughts or feelings of obsessive neurosis. However, contrary to the common view that anxiety was both pathological and morally reprehensible, indicative of a person’s weak will and cowardice, Morita maintained that all the *shinkeishitsu* anxieties originated from a fundamental anxiety that was normal and integral to humanity, which he called “the fear of death.” This, along with “the desire for life,” Morita’s term for the most fundamental desire, were two sides of the same coin—two different aspects of the same psychic force driving people toward self-preservation and self-realization. The stronger the desire for life, the stronger the fear of death, and vice versa (Morita, 1974b, pp. 173–185). Morita contended that *shinkeishitsu*, as a personality type, was primarily characterized by a strong desire for life, that is, a strong fear of death. The *shinkeishitsus*, therefore, suffered not from the inadequacy of nervous or psychic force but its abundance (Morita, 1974b, p. 159; 1974e, pp. 23–24).

Hence, similar to Sigmund Freud’s theory of actual neurosis, Morita emphasized the intimate relationship between anxiety and desire (Hartocollis, 2002; MacMillan, 1976). However, instead of sexual desire, Morita conceived the most fundamental desire, that is, the desire for life, as an instinctive force that naturally assumed the form of the desire for work and related matters, such as power, dominance, superiority, perfection, and personal advancement (Morita, 1974e, pp. 113–114; 1974g, pp. 216–217). He contended that these were all natural desires, arising spontaneously in response to circumstances; however, the *shinkeishitsus* had lost contact with them for being trapped by intellectual desires shaped by extraneous man-made ideas and norms. The primary goal of work therapy, Morita insisted, was not to train the clients into a good worker through repetitive, mechanical practice, but to help them recover their natural desires for work through immersing themselves in immediate situations, which Morita intended to achieve with the specific arrangement of therapeutic settings and procedures (Fujita, 1986, pp. 198–217).

As stated above, Morita therapy was divided into four stages. Although clients appeared to not engage in substantial work in the first two stages, they were crucial for sharpening clients’ senses and bringing about their awareness of their inner desire for work. In the first stage, new clients were mandated absolute isolation and bed rest and were asked not to alleviate their symptoms by any means. This allowed them to observe for themselves the natural course of their “symptoms” and emotions, which, without deliberate intervention, always declined after reaching peak intensity. Moreover, placed in such circumstances, Morita noted that clients usually felt a great deal of boredom and anxiousness to go outside and work. Morita attributed this feeling of boredom to the awakening of clients’ desire for work and interpreted it as an indication of their readiness for the next stage. Idling all day and doing nothing productive was contrary to human nature and the most painful thing in the world. This was the truth that his clients would come to understand through their experiences (Morita, 1974b, pp. 348–353; 1974e, p. 383).

In the second stage, clients were not allowed to stay in their rooms or idle around during the day. Apart from that, they were free to find and perform any less physically demanding household tasks of their interest. This allowed clients coming out of bed rest to recuperate and, more importantly, created circumstances that would redirect their attention away from themselves and toward the outside world. With heightened senses and renewed interest in their surroundings, they would increasingly feel the desire to work and become anxious to take on more substantial tasks, upon which they would enter the next stage of proper work therapy (Morita, 1974b, pp. 353–357).

Unlike conventional work therapy, Morita did not arrange or assign particular tasks; rather, he simply instructed them to pay constant attention to their surroundings to identify the many tasks requiring completion in a household. Morita valued spontaneity and creativity of work and allowed his clients to perform household tasks of their choice and using their own methods. However, he placed the greatest emphasis on the experience and realization of the instinctive desire for work, reiterating that through work, clients would feel constant anxiety and the desire to do more and achieve better—an essential feature of *shinkeishitsu*. Experiencing this natural “anxiety to work” (*haraharasuru*), which had previously been displaced by intellectual obsessions, was key to the *shinkeishitsus*’ recovery. It was also vital to the enhancement of work efficiency, a fact which Morita criticized the objective sciences of work for having overlooked (Morita, 1974e, pp. 629–630). If clients felt tired and lacked interest in work, Morita would urge them to nonetheless find and stick to a task, during which their interest and desire to work would naturally rise again. In Morita’s view, there was no need to set up a rest–work schedule, which was only useful when people were obligated to work. For those working willingly, the most effective way to refresh themselves often was simply switching to another job (Morita, 1974e, pp. 396, 423, 454–456, 574–576). He also noted that although some clients formerly deemed domestic chores lowly and worthless, once they engaged in work, they felt genuine interest, pleasure, and a sense of accomplishment. Such experiences helped them realize their prejudices and the intrinsic value of work itself (Morita, 1974b, p. 357).

Additionally, Morita instructed his clients to respect and follow the logic and laws of emotion at work. Instead of trying to regulate their emotions according to intellectual and moral ideals, they should be open to all feelings arising during work, including negative feelings such as weariness and tedium. He emphasized that they should not blame themselves for such feelings on moral grounds; rather, they should acknowledge them as an inevitable part of human nature and accept them as they were. Sometimes, individuals may even turn negative feelings into positive motivations. For instance, prompted by the feeling of tedium, they may invent more efficient ways of doing their jobs (Morita, 1974d, pp. 88, 115; 1974e, pp. 408–409, 625–626).

Hence, Morita focused his theory and practice of work therapy on subjective feelings and interests. For *shinkeishitsu* clients obsessed with norms and ideals dictated by contemporary work culture, his work therapy intended to provide a pure experience of work through which they could obtain an intuitive understanding of their innate desires and anxiety toward work and accommodate the natural complexity of feelings associated with work. Moreover, the sense of accomplishment and self-worth that accompanied task completion would enhance their confidence in realizing their desires in the real world. Therefore, Morita therapy enabled clients to place themselves at the center of their working life. This self was not the egoistic and materialistic one of capitalist culture or the moral one of self-cultivation culture but a working self who was constantly anxious, eager to work, and always endeavored to do their best.

Meanwhile, Morita drew attention to the situational and social aspects of work as well, toward which he urged his clients to take a proactive, rather than a merely adaptive, approach by recognizing them as an intrinsic part of work itself. Morita emphasized that one needed to “get into” (*narikiru*) and “become one with” the work situation, that is, to fully engage and identify with the task and all related natural, interpersonal, and social conditions to acquire intimate and full knowledge of how to deal with it (Morita, 1974d, pp. 157, 166–167; 1974e, pp. 384–388). For example, he pointed out that one was able to properly care for a plant only if one had sufficient knowledge about the plant and considered all aspects of its surroundings, including the weather, its symbolic significance, its place in the domestic landscape, and the preferences and feelings of other household members (Morita, 1974d, pp. 134–135, 145, 161–162). Such pragmatic views of work proved both appealing and useful to the *shinkeishitsus* who had found it difficult to adapt to the workplace.

## Work, Self, and Society

Morita posited that for the *shinkeishitsus*, participating in a real work environment was far better training than occupational practice in therapeutic settings (Morita, 1974a, pp. 431–432; 1974e, pp. 203–204). When his clients were hesitant about returning to work, Morita always advised them to stick to their jobs or their disease would never be cured (Morita, 1974a, pp. 595–596; 1974e, pp. 38–41, 137, 190, 341). In Morita’s view, *shinkeishitsu* originated from one’s unrealistic, egotistical desires, which could only be overcome through experiential learning about the dynamic relationships between the self and the outside world in real work situations.

This is reflected in his theory and treatment of writer’s cramp (*shokei*). Morita contended that writer’s cramp was a symptom of *shinkeishitsu*, rather than a neuromuscular disorder. Like other *shinkeishitsu* symptoms, it resulted from the *shinkeishitsus* paying undue attention to handwriting difficulties naturally occurring in certain conditions. If left alone, they would disappear. However, the *shinkeishitsus* attempted to fix them by trying various writing postures, which further distorted their movements, enhanced their sensitivity, and worsened and perpetuated the problems. Morita explained the *shinkeishitsus’* obsession in terms of their desire for perfect handwriting. This, in turn, reflected their egotistical tendency and disregard for the original purpose of writing, which was to communicate. To remedy this obsession, Morita contended that patients must stop attempting to find a comfortable writing posture or practicing handwriting. Instead, they would only write when the need arose in real-life situations. When they felt the need to communicate, they, naturally, would write carefully and meticulously to make their handwriting easy for others and themselves to read, regardless of its appearance or their feelings of awkwardness. Morita noted that by adopting such a pragmatic attitude toward writing, most of his clients with writer’s cramp had been cured, which was evident from the visible improvement in their handwriting (Morita, 1974c, pp. 137–157; 1974e, pp. 38–41, 62–63).

The same principle was applied to the treatment of erythrophobia (*sekimen kyōfushishō*). Morita initially regarded erythrophobia as an incurable disease; however, he later found that it responded well to his treatment program. In his view, erythrophobia was also obsessive in nature and caused by the “psychological interaction” of patients’ attention and sensitivity to their blushing faces. Ultimately, it originated from their desire to impress and stand out among others. Therefore, the condition was usually aggravated or only occurred when they met people they were eager to impress. Morita considered this desire for attention normal and natural and stated that it led to erythrophobia in the *shinkeishitsus* only because of their egotistical tendencies. He emphasized that patients with erythrophobia must learn to focus attention on the person whom they wish to impress rather than on themselves. For example, in conversations with superiors at the workplace, instead of monitoring and trying to avoid blushing, they must be fully engaged in the conversation. They should try their best to understand the topics and carefully observe, empathize, and respond to superiors’ needs and concerns. By pursuing their desire more productively, they would be able to find a dynamic balance between desire and anxiety and free themselves from the futile obsession with blushing (Morita, 1974d, pp. 143–146, 232–234, 448–449; 1974e, pp. 280–284, 525–528, 618–621, 712–713).

Hence, for the treatment of writer’s cramp and erythrophobia, Morita underscored the importance of turning attention away from oneself and focusing on the actual interpersonal and social situations. He offered similar advice to clients troubled by relationship problems in their families and working lives. Many of Morita’s clients experienced difficult relationships at home or the workplace, most of which involved conflict with authority figures or the patriarchal order, such as young students at war with their fathers over career choices, housewives disagreeing with husbands over domestic issues, younger sons resenting their eldest brothers’ privileged status in the family, and subordinate employees at odds with their superiors at work. Essentially, Morita’s advice for those clients was that they should not stick to their opinions but rather adopt a flexible and compliant attitude to adapt to the situations. He pointed out that because of their excessive self-consciousness, the *shinkeishitsus* often lacked empathy and flexibility and experienced conflict with authority figures and the established social order. To resolve these conflicts and overcome their egocentric proclivities, Morita emphasized the importance of compliance and adaptability. He taught the *shinkeishitsus* that they must learn to be humble and recognize the experience, knowledge, and social positions of their seniors and the accumulated wisdom of customs and traditions. Fundamentally, just as they were expected to comply with the nature and reality of the mind, they were urged to learn and comply with the quasi-natural principles governing interpersonal and social relationships that underpin social orders. This would enable them to adapt to and handle any life or work situation without conflict (Morita, 1974a, pp. 413–423; 1974b, pp. 201–219; 1974e, pp. 44–46, 62–75, 568–571, 748–749; 1974g, pp. 269–271).

This seemingly conservative stance on the conflict between individuals and authorities may be why historians and contemporary Morita therapists have paid little attention to Morita’s social thought, excusing it as a product of his time (Aoki, 1974, p. 774; Kumano, 1974, pp. 844–845). However, Morita made a clear distinction between true compliance and blind subservience and emphasized the primary importance of the self. He described people of a servile nature as *otsukai konjō,* those who would abandon their ideas and blindly follow superiors’ orders. They did not really engage in work, had little spontaneity and creativity, and merely did what they were told thoughtlessly and carelessly (*yattsuke shigoto*). By contrast, those with a truly compliant attitude maintained their views even when they carried out their superiors’ orders. They remained skeptical, complying with the orders while reflecting on the outcomes to evaluate their accuracy, which would become apparent to both them and their superiors. Most importantly, despite differences in opinion, they brought their full selves to work and exerted their creativity to fulfill the tasks. Morita likened this compliant attitude to the attitude demanded of his clients in therapy: regardless of their doubts about the effectiveness of Morita’s therapy, they were expected to abide by the rules and be fully engaged in the treatment process; ultimately, their experience would inform them of its effectiveness or lack thereof. Morita insisted that there would be no true compliance without opposition between oneself and others. He contended that the sense of self was a natural feeling arising from interactions with the environment, which the common moral education and self-cultivation teachings had been wrong in exhorting people to denounce or suppress. Instead, one must always put oneself at the forefront (*ga wo ositateyo*) and work hard to seek self-improvement and self-realization akin to all great people (Morita, 1974d, pp. 133–135, 1974e, pp. 76–77, 186, 266–268, 509–510, 556–557, 660–662, 684–686).

Hence, concerning the tension between the self and society, Morita did not prescribe a conformist attitude of obedience and submission but highlighted the importance of transcending one’s subjective limitations by accommodating and incorporating other—objective, social, and interpersonal—perspectives. This view was appealing and effective for his mostly middle-class clients and readers who, with a strong sense of self and steeped in the contemporary culture of self-advancement and self-improvement, found it hard to adapt to and advance themselves in their contemporary work environment. Emphasizing spontaneity and creativity, as well as compliance and adaptability, Morita’s teachings helped them find a balance between the self and society. Meanwhile, Morita exalted the contribution of the *shinkeishitsus* to a society’s stability, prosperity, and progress. He called them “the class of *kōshin* (stability)” or “*kōsan* (fixed property),” in contrast to that of *kyoei* (vanity) or *tōshi* (investment), which was characterized by a materialistic worldview and life philosophy of both the capitalist and the proletariat classes. He contended that while the second was a dangerous source of social conflict and unrest for the blind and unreflective pursuit of self-interest and material desires, the first constituted a stable yet dynamic core of society by constantly working hard to improve and realize themselves in real life (Morita, 1974g, pp. 262–278).

## Conclusion

Although contemporary practitioners often highlight the cultural features of Morita therapy, its social and historical character is equally important for understanding its power and appeal during Morita’s time. Morita conceptualized *shinkeishitsu* as a disease of innate temperament and a social illness. The *shinkeishitsus*’ suffering was driven as much by the contemporary work environment and ideologies as by their personality traits. As a core element of an individual’s identity and relationships, work was an important concern and played a central role in the development of his clients’ “symptoms.” For their work-related obsessions, the self-cultivation methods and the conventional models of work therapy provided no remedy, as they, too, were based on the prevailing idealized views of work. Focusing on the subjective emotional experience of work, Morita’s treatment program led clients to place themselves at the center of their working life. Moreover, for his mostly middle-class clients and readers, Morita’s reconciliation between the self and society and between autonomy and compliance was persuasive and useful, providing a work philosophy and worldview whereby they could integrate themselves into the work environment without losing self-worth. Therefore, Morita’s social and cultural critiques were not ideologically driven and constituted an integral part of his *shinkeishitsu* theory and therapy.

Understanding the social and historical nature of Morita’s original therapy may have implications for contemporary Morita therapists. Concerning the difficulties currently faced by Morita therapy, some therapists have noted that increasingly fewer people have the typical *shinkeishitsu* personality, which, they believe, is attributable to the impact of changing familial, social, and cultural environments on one’s personality (Fukazawa, 1996; Nakamura, 1996; Ushijima, 1996, 1997; Watanabe, 1996). This, along with practical difficulties, poses an existential threat to Morita therapy. However, regardless of the number of *shinkeishitsus*, it is important to note that the manifestations of their obsessive tendencies should have transformed along with society and selfhood. Morita’s understanding of the *shinkeishitsus*’ desire and anxiety is both general and specific, as well as ontological and situational. The *shinkeishitsus* may become less concerned about work but have the same strong “desire for life” and “fear of death.” Work, obviously, is no longer as important an object of desire and anxiety as it was in pre-war Japan. Work-related issues and virtues of paramount concern to Morita’s clients, such as personal advancement, work efficiency, workplace relationships, fatigue, and diligence, have lost much of their relevance. Instead, in most advanced societies, emotions, feelings, and happiness have become the greatest objects of desire and anxiety, with concepts, virtues (and vices), and ideals constructed around them becoming the new targets of obsession. With his theory of *shinkeishitsu*, Morita recast neurasthenia, a disease of overwork that was characteristic of his time, as *shinkeishitsu*, a disease of obsession with work. Although neurasthenia has long since disappeared, it is worth exploring whether Morita’s insights could contribute to understanding and remedying the mental disorders of our era.

## Footnotes

1 *Shinkeishitsu* (literally, nervous temperament) is a concept that Morita proposed to replace “neurasthenia.” Morita used this term in three different but related senses. First, it referred to an innate temperament or personality type, characterized by strong hypochondriacal tendencies. Second, it denoted a specific mental disorder caused by this innate temperament, which Morita further divided into three subtypes: simple *shinkeishitsu*, paroxysmal neurosis, and obsessive neurosis (Morita, 1998). Third, Morita often used this term to refer to people with *shinkeishitsu* temperament or experiencing the *shinkeishitsu* disorder. To avoid repetition, this study will use “*shinkeishitsus*” to refer to both the temperament and the disorder of *shinkeishitsu* when the connotations of the term are sufficiently clear from the context and use “the shinkeishitsu” to refer to people with *shinkeishitsu*, similar to the usage of “the neurotics” in English.

2 These symptoms included the physical and mental discomfort of simple *shinkeishitsu*, the sudden outburst of intense fear of paroxysmal neurosis, and the recurring anxiety-provoking ideas of obsessive neurosis (Fujita, 1986: 90–105).

3 Keigai is Morita’s pseudonym, which literally means “beyond appearance.”
